# Effect of Cutaneous Feedback on the Perception of Virtual Object Weight during Manipulation

**DOI:** 10.1038/s41598-020-58247-5

**Published:** 2020-01-28

**Authors:** Jaeyoung Park, Bukun Son, Ilhwan Han, Woochan Lee

**Affiliations:** 1 Korea Institute of Science and Technology, Robotics and Media Institute, Seoul, 02792 South Korea; 2 Seoul National University, Department of Mechanical Engineering, Seoul, 08826 South Korea; 3 Incheon National University, Department of Electrical Engineering, Incheon, 22012 South Korea

**Keywords:** Electrical and electronic engineering, Mechanical engineering

## Abstract

Haptic interface technologies for virtual reality applica have been developed to increase the reality and manipulability of a virtual object by creating a diverse tactile sensation. Most evaluation of the haptic technologies, however, have been limited to the haptic perception of the tactile stimuli via static virtual objects. Noting this, we investigated the effect of lateral cutaneous feedback, along with kinesthetic feedback on the perception of virtual object weight during manipulation. We modeled the physical interaction between a participant’s finger avatars and virtual objects. The haptic stimuli were rendered with custom-built haptic feedback systems that can provide kinesthetic and lateral cutaneous feedback to the participant. We conducted two virtual object manipulation experiments, 1. a virtual object manipulation with one finger, and 2. the pull-out and lift-up of a virtual object grasped with a precision grip. The results of Experiment 1 indicate that the participants felt the virtual object rendered with lateral cutaneous feedback significantly heavier than with only kinesthetic feedback (*p* < 0.05 for *m*_*ref*_ = 100 and 200 g). Similarly, the participants of Experiment 2 felt the virtual objects significantly heavier when lateral cutaneous feedback was available (*p* < 0.05 for *m*_*ref*_ = 100, 200, and 300 g). Therefore, the additional lateral cutaneous feedback to the force feedback led the participants to feel the virtual object heavier than without the cutaneous feedback. The results also indicate that the contact force applied to a virtual object during manipulation can be a function of the perceived object weight *p* = 0.005 for Experiment 1 and *p* = 0.2 for Experiment 2.

## Introduction

The recent growth of the virtual reality industry calls for the means to let a user vividly feel and naturally interact with the virtual environment. In this context, various haptic technologies have been developed to increase the reality and manipulability of a virtual object by creating a diverse tactile sensation. A typical way of providing a tactile sensation to the user is kinesthetic feedback, which applies an interaction force between the user and a virtual object with a robot arm, called a force feedback interface. Most of force feedback interfaces allow the user a 3-degree-of-freedom (3DOF) or 6-degree-of-freedom (6DOF) motion and provide kinesthetic feedback to a tool or a stylus/stylus that s/he is holding during virtual interaction^1–4^. A specific form of the force feedback interface is a glove-type or exoskeleton force feedback interface, which can represent contact force to the user’s fingertips^5–9^. This type of haptic interface has the advantage of letting the user directly feel the contact at his/her fingers while minimizing the constraint on hand movement. Another form of haptic interface is a cutaneous or fingertip haptic interface that stimulates the user’s skin at the fingertip^10^. This type of haptic device is developed to emulate the sensation of touching a real object by stimulating RA, SA1, and SA2 type mechanoreceptors^11–14^. Thus, tactile cues imparted from a cutaneous interface include contact location information^15–17^, the orientation of contact area^18,19^, and the change of contact area^20,21^. Most of the aforementioned haptic feedback interfaces have been evaluated in terms of improving the perception of a static virtual object’s tactile properties. On the contrary, little is known about the effect of haptic feedback on the tactile perception during object manipulation.

There are theoretical and experimental grounds that the addition of cutaneous stimulus can lead to a change in the perception of an object’s physical properties. According to the studies on multi-modal sensory integration, the addition of sensory cue with different modality can result in the shift of percept when the different types of information integrate optimally. Such optimal sensory integration is found in various combination of sensory cues, e.g., visual-haptic^22–24^, intra-visual^25^, or audio-visual integration^26,27^. Narrowing the range of modalities to only haptic cues, additional cutaneous feedback, along with kinesthetic cues, are known to affect the haptic perception of physical properties such as friction^28^ or surface stiffness^21,29^ or object size^30^, as well as hand kinesthesia^31,32^. However, fewer studies have been conducted to evaluate the relative role of cutaneous information in object weight perception, which is a crucial component in manipulation^33–35^.

Multiple studies indicate that lateral cutaneous feedback at fingertip provides information on an object’s weight. When one lifts an object with a precision grip, tangential torque is applied to fingertip skin depending on object weight^36–38^. Noting that the load torque is a function of object weight, a strong correlation is expected between weight perception and tangential torque at the fingertip. Also, cutaneous feedback to the fingertip is known to create an illusory perception of force with or without kinesthetic feedback^39–42^. Considering its strong correlation to force sensation, we can expect weight perception is affected by cutaneous feedback. The focus of the studies above was mainly on either the relation between object weight and tangential torque profile or the effect of lateral skin stretch feedback that substituted kinesthetic feedback. In the meanwhile, it is hard to find studies on the relative role of lateral cutaneous feedback on object weight perception during manipulation in the view of multi-modal sensory integration.

**Figure 1:**
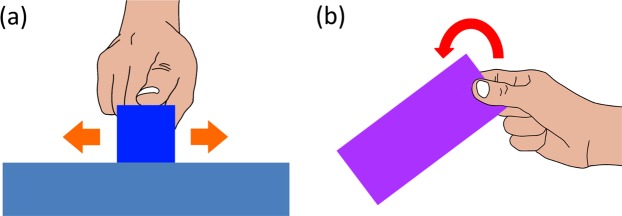
Description of experimental tasks to evaluate the effect of cutaneous feedback on the perception of virtual object weight. (**a**) Lateral manipulation of a virtual object on the floor with one finger, and (**b**) Lifting a virtual object grasped with a precision grip.

This study explores the extent that additional lateral cutaneous feedback to kinesthetic feedback affects the perception of virtual object weight during manipulation. Previous studies on haptic feedback mainly focused on its effect on haptic perception of static object’s surface properties. On the other hand, the present study analyzes the effect of cutaneous feedback on the weight perception during object manipulation. If we assume that the tactile information from cutaneous and kinesthetic feedback integrate optimally, rendering both cutaneous and kinesthetic feedback can shift the perceived object weight from the one represented only with kinesthetic feedback. We, therefore, hypothesize that *the perception of a virtual object weight during manipulation can be affected by the addition of lateral cutaneous feedback*. Also, varying the rate of providing cutaneous feedback may affect the perception of virtual object weight. We tested our hypothesis by evaluating the effect of cutaneous feedback on the perceived weight of a virtual object. The experimental task is to judge the perceived weight of a virtual object, manipulated with one or two fingers (Fig. 1). A participant of the experiment could feel the weight of the virtual object rendered with both lateral cutaneous feedback and kinesthetic feedback. Then, we evaluated the effect of the additional lateral cutaneous feedback on the perceived weight. The next section describes the experimental setup for virtual object manipulation and weight perception.

## Methods

The experimental tasks of the present study were to move a virtual object with an index finger laterally, and to pull-out and lift-up a virtual object grasped with a precision grip. Since tactile information imparted to a user differs by the type of manual task, we built two different experimental setups. This section describes the system overview and haptic rendering algorithm for the two experiments. Then, we illustrate the experimental paradigm and procedure to evaluate the effect of lateral cutaneous feedback on the perceived object weight.

All methods were previously approved by the KIST (Korea Institute of Science and Technology) Institutional Review Board (IRB approval No. 2019-007) and carried out in accordance with the Declaration of Helsinki for research involving human subjects. Informed written consent was obtained from all participants involved in the experiments.

### Experiment 1: rendering haptic feedback during virtual object manipulation with one finger

**Figure 2:**
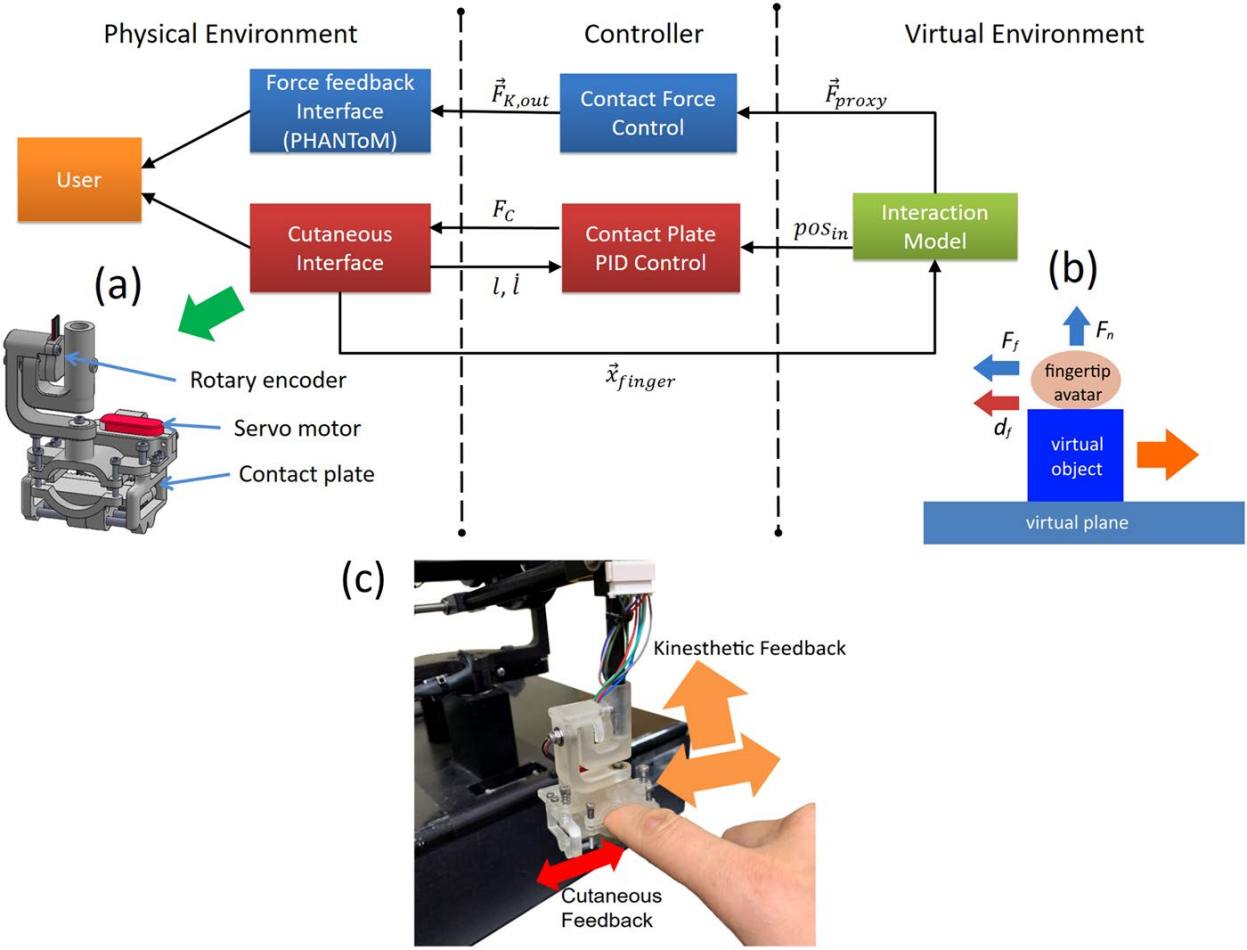
The haptic system architecture for Experiment 1. The interaction force between the user’s fingertip avatar and virtual environment is calculated with a virtual interaction model, including the lateral friction (Virtual Environment). Next, the controller transfers adjusted stimuli values to the haptic interface (Controller). Then, a user can perceive the haptic stimuli from the haptic interface providing lateral cutaneous and kinesthetic feedback (Physical Environment). (**a**) A cutaneous interface renders lateral friction by moving a contact plate placed beneath the user’s fingertip. (**b**) The virtual interaction model calculates the contact force (***F***_*n*_ and ***F***_*f*_) and cutaneous feedback (*d*_*f*_). (**c**) Experimental apparatus to provide the cutaneous and kinesthetic feedback to a participant’s fingertip during object manipulation with an index finger.

For the experiment with one finger object manipulation in Fig. 1(a), we set up a virtual environment where a user can manipulate a virtual cube with one finger by feeling the contact force with a haptic system. Figure 2 shows the overall experimental system architecture, including the virtual environment, controller, and physical environment. The haptic interface consists of a force feedback interface and a custom-built cutaneous feedback interface (Fig. 2(a)). The cutaneous feedback interface is worn around the user’s index fingertip beneath which a contact plate is located. When the user laterally moves a virtual object, the fingertip skin is deformed by moving the contact plate with a servo motor (model HV75K, MKS Servo Tech, Ilan, Taiwan). The rotation of a joint of the cutaneous interface or the motor is tracked with a magnetic rotary encoder (RM08, RLS, Slovenia). Being attached to the force feedback interface, the user can feel both cutaneous and kinesthetic feedback simultaneously.

During the experiment, a user’s fingertip is mapped to a virtual environment and represented as a fingertip avatar (Fig. 2(b)). When the fingertip avatar touches a virtual object, the normal contact force ***F***_*n*_ is calculated based on a virtual proxy based haptic rendering model^43,44^ as follows:

1 $${{\boldsymbol{F}}}_{n}=K({{\boldsymbol{x}}}_{p}-{{\boldsymbol{x}}}_{f}),$$

where *K*, ***x***_*p*_, and ***x***_*f*_ denote virtual object surface stiffness, fingertip proxy contact position, and the fingertip contact position, respectively. Haptic rendering of the lateral friction force is calculated from a contact model based on a Coulomb friction law. A direct application of the Coulomb friction model results in a sudden change of friction force and thus can cause the haptic feedback system unstable. Thus, we modeled the state of static contact and sliding by considering the Coulomb friction model and the velocity of the fingertip based on the piecewise linear approximate Coulomb friction model^45,46^. We calculate the relative lateral speed of fingertip ***v***_*fo*,*t*_ to the virtual object at timestamp *t* as

2 $${{\boldsymbol{v}}}_{fo,t}={{\boldsymbol{v}}}_{f,t}-{{\boldsymbol{v}}}_{o,t-1},$$

where ***v***_*f*,*t*_ and ***v***_*fo*,*t*−1_ are fingertip speed at timestamp *t*, and virtual object speed at timestamp *t − 1*, respectively. By incorporating the Eqs. 1 and 2 with a piecewise linear approximate Coulomb friction model, the following friction state model is derived as:

3 $${{\boldsymbol{F}}}_{f}=\left\{\begin{array}{lll}-{\mu }_{a,s}|{{\boldsymbol{F}}}_{n}|\frac{{{\boldsymbol{v}}}_{fo,t}}{{v}_{th}} & |{{\boldsymbol{v}}}_{fo,t}| < {v}_{th} & ({\rm{Static}}\,{\rm{Contact}})\\ -{\mu }_{a,d}|{{\boldsymbol{F}}}_{n}|sgn({{\boldsymbol{v}}}_{fo,t}) & {\rm{otherwise}} & ({\rm{Sliding}})\end{array}\right.,$$

where ${\mu }_{a,s}$, ${\mu }_{a,d}$, and ${v}_{th}$ are static and dynamic friction coefficients between the virtual fingertip, and state switching threshold speed, respectively. We modeled the tangential force between the virtual object and the virtual surface based on the Coulomb friction law as follows:

4 $${{\boldsymbol{F}}}_{o}=\left\{\begin{array}{ll}-{{\boldsymbol{F}}}_{f} & {\rm{if}}\,{{\boldsymbol{v}}}_{O}=0\,{\rm{and}}|{{\boldsymbol{F}}}_{f}|\le |{\mu }_{b}({m}_{obj}{\boldsymbol{g}}-{{\boldsymbol{F}}}_{n})|\\ -|{\mu }_{b}({m}_{obj}{\boldsymbol{g}}-{{\boldsymbol{F}}}_{n})|sgn({{\boldsymbol{F}}}_{f}) & {\rm{otherwise}}\end{array}\right..$$

where *m*_*o*_*bj* is the virtual object weight. Equations 3 and 4 decide whether the virtual objects moves or not. When the virtual object moves, its position is updated along with its speed. The force to be exerted by the force feedback, ${{\boldsymbol{F}}}_{kin}={{\boldsymbol{F}}}_{n}+{{\boldsymbol{F}}}_{f}$ is a vector summation of the lateral and normal contact force, calculated as

5 $${{\boldsymbol{F}}}_{kin}={{\boldsymbol{F}}}_{n}+{{\boldsymbol{F}}}_{f}.$$

On each servo loop, the calculated lateral contact force ***F***_*kin*_ is fed to the controller and the force feedback interface. The desired displacement of the contact plate on the cutaneous interface is calculated as ${d}_{f}={l}_{f}|{{\boldsymbol{F}}}_{f}|sgn({{\boldsymbol{F}}}_{{\boldsymbol{f}}})$. Then, the contact plate is positioned to the desired displacement with a PID controller. The output torque from the controller is converted to a PWM signal generated by a multifunction board (Model 826, Sensoray Co. Inc., OR, U.S.A) and is sent to a motor controller, connected to the servo motor of the cutaneous interface.

Figure 2(c) shows the experimental apparatus that can render both lateral cutaneous feedback and force feedback, as a user moves a virtual object with an index finger. The interaction force due to the friction between a fingertip avatar and a virtual object was rendered for force and cutaneous feedback (Fig. 2(c)). The force feedback interface directly applied the calculated force ***F***_*kin*_ with a commercially available force feedback interface (PHANToM Premium 1.0, 3D Systems, SC, USA). The custom-built haptic interface (Fig. 2(a)) rendered lateral cutaneous feedback by moving a contact plate placed beneath a participant’s fingertip.

### Experiment 2: rendering haptic feedback during virtual object manipulation with a precision grip

**Figure 3:**
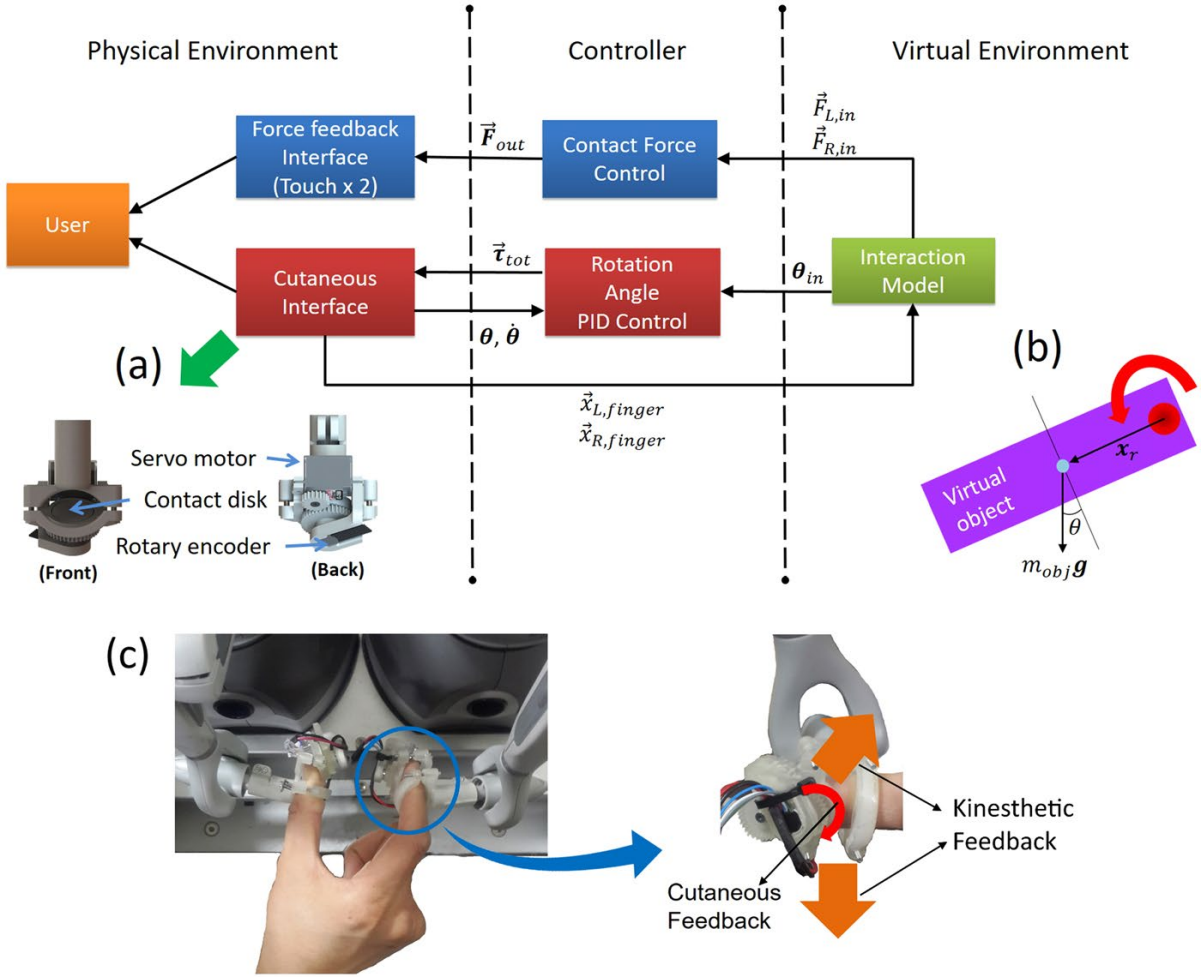
The haptic system architecture for Experiment 2. The interaction force between the user’s fingertip avatars and virtual environment is calculated with a virtual interaction model, including the torsional friction (Virtual Environment). Next, the controller transfers adjusted stimuli values to the haptic interface (Controller). Then, a user can perceive the haptic stimuli from the haptic interface providing torsional cutaneous and kinesthetic feedback (Physical Environment). (**a**) A cutaneous interface renders torsional friction by moving a contact disk touching the user’s fingertip. (**b**) The virtual interaction model calculates the contact force (***F***_*kin*_) and the cutaneous feedback generated by rotating the contact disk. (**c**) Experimental apparatus to provide the cutaneous and kinesthetic feedback to a participant’s fingertip. A contact plate on the cutaneous interface rotates to provide torsional friction feedback to the fingertip as the virtual object rotates.

For the experiment with two-finger object manipulation in Fig. 1(b), we set up a virtual environment where a user can feel when lifting a virtual cuboid grasped with a precision grip. Figure 3 shows the overall haptic system architecture, including virtual environment, controller, and physical environment. Two force feedback interfaces provides a user with kinesthetic feedback as s/he grasps a virtual object with a precision grip. A cutaneous feedback interface (Fig. 3(a)) was attached to the end-effector of each force feedback interface and a contact skin rotates fingertip skin, which emulates the torsional skin deformation as an object rotates due to its weight. A magnetic rotary encoder (RM08, RLS, Slovenia) with the resolution of +0.3 deg is attached at the bottom of the contact disk to track the disk’s rotation angle. A pair of springs tightly fixes the haptic interface to a user’s fingertip at the distal interphalangeal (DIP) joint.

Haptic rendering of the force and cutaneous feedback for manipulating an object with a precision grip was calculated in a similar way to Experiment 1. When a user holds a cuboid with his/her index finger and thumb (Fig. 3(b)), we can calculate the frictional torque at the surface of the contact point (red circle in the figure) as ${\tau }_{f}=\frac{2}{3}{\mu }_{s}{r}_{disk}|{{\boldsymbol{F}}}_{n}|$ (*μ*_*s*_: friction coefficient, *r*_*disk*_: rotating disk radius, and ***F***_*n*_: normal contact force)^47^. Then, applying the Coulomb friction model^48^ on the torque, we get the friction model as follows:

6 $${{\boldsymbol{\tau }}}_{f}=\left\{\begin{array}{ll}-{{\boldsymbol{\tau }}}_{e} & {\rm{if}}\,\omega =0\,{\rm{and}}|{{\boldsymbol{\tau }}}_{e}|\le \frac{2}{3}{\mu }_{s}{r}_{disk}|{{\boldsymbol{F}}}_{n}|\\ -\frac{2}{3}{\mu }_{s}{r}_{disk}|{{\boldsymbol{F}}}_{n}|sgn({{\boldsymbol{\tau }}}_{e}) & {\rm{otherwise}}\end{array}\right.,$$

where ***τ***_*e*_ and ***x***_*r*_ are the torque due to the object weight and the displacement vector from the contact point to the center of mass, respectively. ***F***_*n*_ is the normal contact force in Eq. 1. Then, total torque at the fingertip contact point ***τ***_*tot*_ is

7 $${{\boldsymbol{\tau }}}_{tot}={{\boldsymbol{\tau }}}_{e}+{{\boldsymbol{\tau }}}_{f}\cdot $$

We can calculate the rotation of the contact plate from ${{\boldsymbol{\tau }}}_{tot}=I{\boldsymbol{\alpha }}$ (***I***: the moment of inertia and *α*: angular acceleration), and *ω* with Euler integration. The rotation of the contact plate is then updated as follows:

8 $${\theta }_{i+1}={\theta }_{i}+\omega \delta t,$$

where *i* denotes the timestamp. The force to be exerted by each force feedback device in the vertical direction is calculated as ${{\boldsymbol{F}}}_{v}=(0.5{m}_{obj}-{m}_{tip}){\boldsymbol{g}}({m}_{obj}:{\rm{virtual}}\,{\rm{object}}\,{\rm{weight}},\,{\rm{and}}\,{m}_{cut}:{\rm{cutaneous}}\,{\rm{feedback}}\,{\rm{interface}}\,{\rm{weight}})$ to avoid the effect of haptic weight interface on the perception of virtual object weight. Then, total force to be exerted by each force feedback interface ***F***_*kin*_ is,

9 $${{\boldsymbol{F}}}_{kin}={{\boldsymbol{F}}}_{v}+{{\boldsymbol{F}}}_{n},$$

where ***F***_*n*_ is the normal contact force calculated by the Eq. 1.

Figure 3(c) shows the experimental apparatus that can provide kinesthetic feedback and cutaneous feedback to the participant. Two commercially available force feedback interfaces (Touch, 3D Systems, SC, USA) directly rendered the force vector ***F***_*kin*_ as a vector sum of normal-directional contact force ***F***_*n*_ and the weight of the virtual cuboid, ***F***_*v*_. A cutaneous interface (Fig. 3(a)) is attached to the end-effector of each force feedback interface to provide cutaneous feedback by rotating contact plate.

### Friction coefficients of real object surfaces

We measured the friction coefficients between real objects to estimate the degree of slipperiness/roughness of the virtual friction coefficient for the experiments. The friction coefficient was measured for three surface materials (metal, plastic, and silicon), and two sliding object materials (metal, and silicon). We employed a typical inclined slope method to measure the static friction coefficient, where the friction coefficient was decided as a tangent of the angle that the object begins to slide. For the sake of measurement accuracy, we attached markers on the sides of the slope, video-recorded the sliding, and derived the sliding angle by analyzing the image at the moment of sliding. The measurement was conducted three times for each condition. The static friction coefficients between two materials were 0.21 ± 0.01 (metal-metal), 0.16 ± 0.03 (metal-plastic), 0.65 ± 0.12 (metal-silicon), 0.43 ± 0.03 (silicon-plastic), and 0.55 ± 0.11 (silicon-silicon).

### Experimental procedure

For the experiments, we employed a one-up one-down adaptive procedure to match the perceived weight of a virtual object rendered with both cutaneous and kinesthetic feedback to that rendered only with kinesthetic feedback^49^. The adaptive procedure estimates the PSE of comparison stimuli that is perceived to be equivalent to the reference stimulus.

Each participant conducted four (six) experimental runs by reference object weight and *l*_*f*_ for Experiment 1 (*μ*_*s*_ for Experiment 2). The order of reference stimuli was randomized for each participant. On each trial, a participant was presented with two virtual objects: a reference object rendered both with cutaneous and kinesthetic feedback and a comparison object rendered only with force feedback. If the participant responded that the reference object felt heavier than the comparison plane, the weight of the comparison plane decreased. Otherwise, the weight of the comparison plane increased. The initial weight was 400 g and 300 g for Experiments 1 and 2, respectively, considering the stability of the force feedback interface used for each experiment. The step size of increasing/decreasing the weight changed from 100 g to 25 g after the first three reversals of the responses. Each experimental run terminated after 12 reversals of the responses at the smaller step size. The total number of trials for one experimental run typically ranged between 30 and 40.

Before the main experiment, a participant was seated in front of a computer and asked to wear noise-canceling headphones (MDR10RNC, Sony, Tokyo, Japan). Then, s/he inserted his/her index finger inside cutaneous interface (Figs. 2(a) and 3(a)). Then, a training session was initiated where the participant could feel the weight of a virtual object rendered with both cutaneous and force feedback or only with force feedback. Virtual fingertips were visually displayed as green spheres along with the virtual object. When the participant felt ready for the main experiment, the training session terminated.

In the main experiment, the order of presenting reference and comparison stimuli was randomly decided on each trial. In the beginning, fingertip avatars and a virtual object were displayed on the screen. In Experiment 1, a participant was asked to laterally move the virtual object with an index finger and feel the object weight. In Experiment 2, a participant was asked to grasp and lift up the cuboid laid on a virtual plane with a precision grip and feel the object weight. During the main experiment, white noise was played on headphones to block possible audio cues from the haptic interface. By hitting the enter key, the participant moved to the next phase to feel the other stimulus. Then, the participant was asked to indicate which stimulus felt heavier. For each trial, the magnitude of comparison stimulus, the answer, collision depth to derive the contact force, and trial time were recorded. After each experimental run, the participant took a 5-min break to minimize the fatigue from the experiment.

## Results

We conducted two psychophysical experiments by how to manipulate the object (Fig. 1), to investigate the effect of the cutaneous feedback on the perceived object weight. In the first experiment, participants moved a virtual object on a floor sideways by feeling cutaneous and kinesthetic feedback, to perceive the object weight. The second experiment evaluated the effect of cutaneous feedback on the perceived weight of a virtual object grasped and lifted with a precision grip. For both experiments, we also investigated how the rate of providing cutaneous feedback affects the perceived weight of the virtual object. The following subsections describe the experimental setup and report experimental results.

### Experiment 1: Perceived weight of a virtual object manipulated with one finger

The goal of this experiment is to evaluate the effect of cutaneous feedback on the perceived weight of a virtual object manipulated with an index finger. Total 12 subjects participated in the experiment which matched the perceived weight of a virtual object rendered both with cutaneous and kinesthetic feedback to the one rendered with kinesthetic feedback only.

In the main experiment, a participant laterally manipulated a virtual cube (dimension: 3 cm × 3 cm × 3 cm) with a weight of 100 and 200 g, which is about as heavy as a hand-held device, with an index finger. We decided the weight of the virtual object to be around that of a handheld device while conserving the stability of the force feedback interface. The friction coefficients were 0.74, 0.57, and 0.25 for fingertip-object static friction (*μ*_*a,s*_), fingertip-object dynamic friction (*μ*_*a,d*_), and object-floor friction respectively (*μ*_*b*_). We intentionally chose the friction coefficients for fingertip-object (∼silicon-metal) to be higher than that for object-floor (∼metal-metal). Then, the object can slide when sufficient normal and lateral force is applied. For the rendering of cutaneous feedback, we define *l*_*F*_, which is the ratio of contact plate displacement to the lateral force. We used two *l*_*F*_ values, 0.005 and 0.01 m/N for the experiment to evaluate the effect of cutaneous feedback intensity on the perceived weight of a virtual object.

A one-up one-down adaptive procedure was employed to derive the point of subjective equality (PSE) for a reference stimulus rendered with cutaneous and kinesthetic feedback, matched to the weight of a virtual object rendered only with kinesthetic feedback. Both cutaneous and kinesthetic feedback was provided to a participant for the reference stimuli while only kinesthetic feedback was provided for the comparison stimuli.

**Figure 4:**
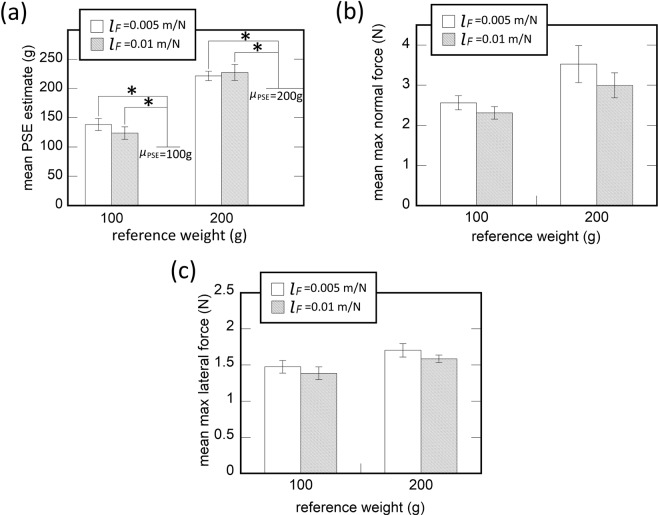
The results of Experiment 1. (**a**) Mean estimated PSE of perceived virtual object weight by *l*_*F*_ and reference weight. (**b**) Mean of the maximum normal-directional contact force by *l*_*F*_, and reference weight. (**c**) Mean of the maximum lateral contact force by *l*_*F*_, and reference weight. Error bars indicate standard errors.

Figure 4(a) shows the average PSE estimate of a virtual object weight plotted as a function of reference object weight by two *l*_*F*_ values. We compared the PSE estimate to the reference object weight, to see the effect of cutaneous feedback on the perceived object weight. The results of one-sample t-test ($${H}_{0}:{\mu }_{PSE} > {m}_{ref}$$ where *m*_*ref*_ means the reference weight) indicate that the PSE estimates are significantly larger than the reference object weight for all experimental conditions [$$t(11)=3.73,p=0.0017$$ for *l*_*F*_ = 0.005 m/N, *m*_*ref*_ = 100 g; $$t(11)=2.69,p=0.011$$ for *l*_*F*_ = 0.005 m/N, *m*_*ref*_ = 200 g; $$t(11)=2.23,p=0.023$$ for *l*_*F*_ = 0.01 m/N and *m*_*ref*_ = 100 g; $$t(11)=2.0,p=0.035$$ for *l*_*F*_ = 0.01 m/N, *m*_*ref*_ = 200 g]. Thus, the cutaneous feedback to the fingertip led the participants to feel virtual object weight heavier than when there is only kinesthetic feedback. When we conducted a two-way repeated measures ANOVA on the PSE estimates with the factors of reference weight and *l*_*F*_, only *m*_*ref*_ had a significant effect on the PSE estimates [$$F(1,11)=0.223,p=0.646,{\eta }^{2}=0.02$$ for *l*_*F*_; $$F(1,11)=90.95,p < 0.001,$$$${\eta }^{2}=0.892$$ for *m*_*ref*_]. No significance interaction between the two factors was found [$$F(1,11)=1.447,$$$$p=0.254,{\eta }^{2}=0.116$$]. Overall, the addition of lateral cutaneous feedback led the participants to feel a virtual object heavier than the one rendered with only kinesthetic feedback. The modulation of *l*_*F*_ did not affect the perception of virtual object weight.

Figure 4(b) shows the mean of the maximum normal-directional contact force by *l*_*F*_ as a function of reference object weight, *m*_*ref*_. We conducted a two-way repeated measure ANOVA to determine the effects of *l*_*F*_ and *m*_*ref*_ on the maximum normal-directional contact force. We found no significant interaction of the two factors [$$F(1,11)=0.31,p=0.589$$, $${\eta }^{2}=0.027$$]. *m*_*ref*_ had a significant main effect on the maximum normal-direction directional contact force [$$F(1,11)=11.94,p=0.005,{\eta }^{2}=0.52$$] meaning increasing trend of the maximum normal-directional contact force with the increase of *m*_*ref*_. In the meanwhile, the effect of *l*_*F*_ was insignificant [$$F(1,11)=1.14,p=0.31,{\eta }^{2}=0.09$$]. In Fig. 4(c), the mean of the maximum lateral contact force is shown by *l*_*F*_ as a function of reference object weight. When we conducted a two-way repeated measure ANOVA with the factors *l*_*F*_ and *m*_*ref*_, no significant two-way interaction was found between the two factors [$$F(1,11)=0.046,$$$$p=0.84,{\eta }^{2}=0.004$$]. The effect of *m*_*ref*_ was significant on the maximum lateral contact force [$$F(1,11)=7.37,$$$$p=0.02,{\eta }^{2}=0.4$$], which implies that the maximum lateral contact force increased with the increase of *m*_*ref*_. *l*_*F*_ had no significant main effect [$$F(1,11)=1.19,p=0.3,{\eta }^{2}=0.111$$].

Overall, the addition of lateral cutaneous feedback to force feedback resulted in the increase of the perceived weight of a virtual object during one-finger manipulation. It is also notable that the reference object weight significantly affected both the normal and lateral force contact force. We discuss the implication of the findings in the discussion section.

### Experiment 2: Perceived weight of a virtual object grasped and lifted with a precision grip

The goal of this experiment is to examine the effect of torsional cutaneous feedback on the weight perception of a virtual object held with precision grip during object pull-out and lift-up task. A new group of 12 subjects participated in the experiment, which matched the perceived weight of a virtual cuboid with the dimension of 6 cm × 5 cm × 14 cm, rendered both with cutaneous and kinesthetic feedback to the one rendered with kinesthetic feedback only. On each trial of the main experiment, a participant was asked to pull out a virtual cuboid attached to a virtual wall, by grasping the object with a precision grip. Then, the virtual object tilted forward, and the participant could feel its weight rendered with kinesthetic and cutaneous feedback.

The weight of the virtual cuboid is 100, 200, and 300 g, which are about as heavy as a hand-held electronics including a smartphone or a tablet. To see the effect of varying cutaneous feedback on the perceived object weight, we used two values for the friction coefficient *μ*_*s*_ between a participant’s fingertip and the cuboid, 0.35 (∼silicon-plastic) and 0.7 (∼silicon-metal). As was in the first experiment, we used a one-up one-down adaptive procedure to derive the point of subjective equality (PSE) for a reference stimulus rendered with cutaneous and kinesthetic feedback, matched to the weight of a virtual object rendered only with kinesthetic feedback.

**Figure 5:**
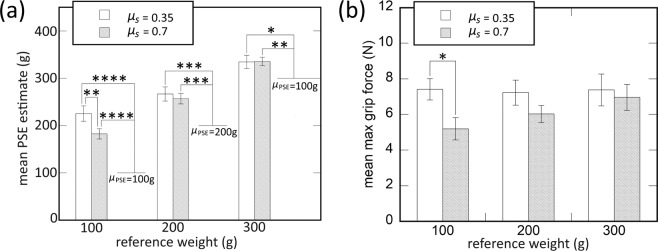
The results of Experiment 2. (**a**) Mean estimated PSE of perceived virtual object weight by *μ*_*s*_ and reference weight. (**b**) Mean of the maximum normal-directional contact force by *μ*_*s*_, and reference weight.

In Fig. 5(a) are shown the average PSE estimates of a virtual object weight plotted as a function of reference object weight by the two *μ*_*s*_ values. When we compared the PSE estimates to the reference object weight with one-sample t-test (${H}_{0}:{\mu }_{PSE} > {m}_{ref}$), the PSE estimates are significantly larger than the reference object weight for all *μ*_*s*_ values and reference weight [$t(11)=7.72,p < 0.0001$ for ${\mu }_{s}=0.02$ and *m*_*ref*_ = 100 g; $t(11)=4.79$ and $p=0.0002$ for ${\mu }_{s}=0.02$ and *m*_*ref*_ = 200 g; $t(11)=2.43$ and $p=0.017$ for *μ*_*s*_ = 0.02 and *m*_*ref*_ = 300 g; $t(11)=7.61$ and $p < 0.0001$ for *μ*_*s*_ = 0.04 and *m*_*ref*_ = 100 g; $t(11)=5.09$ and $p=0.0002$ for ${\mu }_{s}=0.04$ and *m*_*ref*_ = 200 g; $t(11)=2.43$ and $p=0.001$ for *μ*_*s*_ = 0.04 and *m*_*ref*_ = 300 g]. The result is, therefore, consistent with Exp. 1, in that the cutaneous feedback to the fingertip led the participants to feel virtual object weight heavier than when there is only kinesthetic feedback. When a two-way repeated measures ANOVA on the PSE estimates with the factors *μ*_*s*_ and *m*_*ref*_ on the PSE estimates, a significant interaction was found between the two factors [$F(2,22)=3.65,p=0.04,{\eta }^{2}=0.25$]. Simple main effects analysis shows that if *m*_*ref*_ = 100 g, the participants felt the virtual object heavier for *μ*_*s*_ = 0.02 than *μ*_*s*_ = 0.04 [*p* = 0.009]. Overall, the addition of torsional cutaneous feedback led the participants to feel a virtual object heavier than the one rendered with only kinesthetic feedback. The modulation of *μ*_*s*_ affected the perception of virtual object weight when a virtual object light (*m*_*ref*_ = 100 g).

Figure 5(b) shows the mean of the maximum grip force by the type of haptic feedback as a function of reference object weight. When we conducted a three-way repeated measure ANOVA with the factors *μ*_*s*_ and, *m*_*ref*_, on the mean normal-direction contact force, a significant two-way interaction was found [$$F(2,22)=4.69,$$$$p=0.02,{\eta }^{2}=0.3$$]. The result of a simple main effects analysis indicates that there was a significant difference in the maximum grip force between the two *m*_*s*_ values when *m*_*ref*_ = 100 g [*p* = 0.02].

The results of Experiment 2 show a similar trend to those of Experiment 1. Additional torsion feedback led the participants to feel the weight of a virtual object heavier than when there was only kinesthetic feedback, during the pull-out and lift-up task. It is notable that varying *μ*_*s*_ resulted in the change of both perceived weight, and the normal contact force at *m*_*ref*_ = 100 g. We discuss the implications of the findings in the next section.

## Discussion

The results of the experiments show that the addition of cutaneous feedback to kinesthetic feedback during manipulation led participants to feel virtual object weight as being significantly heavier than that rendered with kinesthetic feedback only. We varied the rate of providing cutaneous feedback for the two experiments. We modulated the rate of the contact plate’s lateral motion to the calculated force for Experiment 1. For Experiment 2, we varied the friction coefficient of rotating the contact plate during manipulation. The experimental results indicate that the effect of varying the rate of cutaneous feedback was insignificant except for the reference weight of 100 g in Experiment 2. We also observed that the grip force was affected by the rate of cutaneous feedback for the reference weight of 100 g.

One common feature of the two experiments’s results is that the addition of cutaneous feedback to a participant’s fingertip led the participants to feel a virtual object heavier during manipulation, than with kinesthetic feedback only. This result can be partly explained with the optimal integration model for multi-modal sensory input. Then, the weight perception can be estimated with the following model:

10 $${\hat{S}}_{weight}={w}_{K}{\hat{S}}_{K}+{w}_{C}\hat{C},$$

where ${\hat{S}}_{K}$ and ${\hat{S}}_{C}$ are the estimates of weight from kinesthetic and cutaneous information, respectively, and *w*_*K*_ and *w*_*C*_ are their respective weights. If we assume the distribution of ${\hat{S}}_{C}$ is larger than that of ${\hat{S}}_{K}$, ${\hat{S}}_{weight}$ with the addition of cutaneous feedback will be larger than ${\hat{S}}_{K}$, as is the case with the present study. To substantiate the model, however, additional experimental grounds are needed to show that ${\hat{S}}_{C}$ > ${\hat{S}}_{K}$.

The effect of the friction coefficient on the perceived object weight in Experiment 2 can be explained with the results of previous studies that investigated the perception of fingertip slip perception during tangential loading. Barrea *et al*. demonstrated that the participants of their experiment could perceive the slippage at the fingertip with the partial skin deformation due to passive tangential loading^50^. Similarly, Srinivasan *et al*. showed that the tactile afferents could encode surface slippage at the fingertip^51^. In other words, $\hat{C}$ in 10 is a function of the cutaneous feedback. Then, their results suggest that the cutaneous feedback in the present study generated the tactile information indicating the existence of tangential loading. For Experiment 2, the slippage at the fingertip due to cutaneous feedback could have been more significant for the lower friction coefficient of *μ*_*s*_ = 0.35 because the virtual object would have rotated more easily. In the meanwhile, the difference of the perceived object between the two friction coefficient was insignificant for larger reference weight, *m*_*ref*_ = 200 and 300 g. This result can ascribe to the insensitivity to smaller cutaneous stimulus change as the overall perceived stimulus, ${\hat{S}}_{weight}$ increases with the increase of the reference weight by Weber’s law.

The experimental results suggest that the force applied to virtual object during manipulation can be a function of perceived object weight. The maximum contact force in Experiment 1 shows an increasing trend along with the increase of the perceived weight of the virtual object. Also, when we compare the maximum contact force of Experiment 2 (Fig. 3(b)) to the PSE estimates (Fig. 3(a)), the effect of varying *μ*_*s*_ shows the same trend that the difference is significant for low *m*_*ref*_ value of 100 g. Previous studies^36,38^ indicate that when one is manipulating an object with a precision grip, s/he applies normal force being proportional to tangential torque at the fingertip. Their findings coincide with the results of Experiment 2 where participants felt an object heavier with larger torsional cutaneous feedback (lower *μ*_*s*_) and thus exerted larger grip force. Thus, the contact force during object manipulation can be modeled as a function of the perceived object weight, and thus depend on kinesthetic and cutaneous information as follows:

11 $${F}_{contact}=f({\hat{S}}_{weight})=f({I}_{K},{I}_{C}),$$

where ${\hat{S}}_{weight}$, *I*_*K*_, and *I*_*C*_ are the perceived weight, kinesthetic, and cutaneous information, respectively.

To summarize, the present study provides grounds that the addition of lateral cutaneous feedback to kinesthetic feedback during object manipulation can increase the perceived weight of the object. Also, the experimental results suggest that the contact force during object manipulation can be a function of the perceived object weight.

## References

[1] Massie, T. H. & Salisbury, J. K. The phantom haptic interface: A device for probing virtual objects. In *Proceedings of the ASME winter annual meeting, symposium on haptic interfaces for virtual environment and teleoperator systems*, vol. 55, 295–300 (Citeseer, 1994).

[2] Berkelman, P. J., Butler, Z. J. & Hollis, R. L. Design of a hemispherical magnetic levitation haptic interface device. In *Proceedings of the ASME Winter Annual Meeting*, Symposium on Haptic Interfaces for Virtual Environment and Teleoperator Systems,(Atlanta), vol. 58, 483–488 (1996).

[3] Cholewiak, S. A., Tan, H. Z. & Ebert, D. S. Haptic identification of stiffness and force magnitude. In *2008 Symposium on Haptic Interfaces for Virtual Environment and Teleoperator Systems*, 87–91 (IEEE, 2008).

[4] Lee G, Hur S-M, Oh Y (2016). High-force display capability and wide workspace with a novel haptic interface. IEEE/ASME Trans. Mechatron..

[5] Ryu, D. *et al*. Micro hydraulic system using slim artificial muscles for a wearable haptic glove. In *2008 IEEE/RSJ International Conference on Intelligent Robots and Systems*, 3028–3033 (IEEE, 2008).

[6] Jo I, Bae J (2014). A force-controllable compact actuator module for a wearable hand exoskeleton. IFAC Proc. Vol..

[7] Ben-Tzvi P, Ma Z (2014). Sensing and force-feedback exoskeleton (safe) robotic glove. IEEE Trans. Neural Syst. RehabilitatiEng..

[8] Choi, I., Hawkes, E. W., Christensen, D. L., Ploch, C. J. & Follmer, S. Wolverine: A wearable haptic interface for grasping in virtual reality. In *2016 IEEE/RSJ International Conference on Intelligent Robots and Systems (IROS)*, 986–993 (IEEE, 2016).

[9] Popov D, Gaponov I, Ryu J-H (2016). Portable exoskeleton glove with soft structure for hand assistance in activities of daily living. IEEE/ASME Trans. Mechatron..

[10] Summers IR, Chanter CM (2002). A broadband tactile array on the fingertip. J. Acoustical Soc. Am..

[11] Srinivasan MA, Lamotte RH (1987). Tactile discrimination of shape: responses of slowly and rapidly adapting mechanoreceptive afferents to a step indented into the monkey fingerpad. J. Neurosci..

[12] Srinivasan, M. A. & LaMotte, R. H. *Encoding of shape in the responses of cutaneous mechanoreceptors*, 59–69 (MacMillan Press, 1991).

[13] Johnson KO (2001). The roles and functions of cutaneous mechanoreceptors. Curr. Opin. Neurobiol..

[14] Johansson RS, Flanagan JR (2009). Coding and use of tactile signals from the fingertips in object manipulation tasks. Nat. Rev. Neurosci..

[15] Provancher WR, Cutkosky MR, Kuchenbecker KJ, Niemeyer G (2005). Contact location displays for haptic pereption of curvature and object motion. Int. J. Robot. Res..

[16] Leonardis D, Solazzi M, Bortone I, Frisoli A (2017). A 3-rsr haptic wearable device for rendering fingertip contact forces. IEEE Trans. haptics.

[17] Chinello F, Pacchierotti C, Malvezzi M, Prattichizzo D (2018). A three revolute-revolute-spherical wearable fingertip cutaneous device for stiffness rendering. IEEE Trans. haptics.

[18] Salada, M., Colgate, J. E., Vishton, P. & Frankel, E. Two experiments on the perception of slip at the fingertip. In *Proceedings of the 12th International Symposium on Haptic Interfaces for Virtual Environment and Teleoperator Systems*, 146–153 (2004).

[19] Frisoli A, Solazzi M, Reiner M, Bergamasco M (2011). The contribution of cutaneous and kinesthetic sensory modalities in haptic perception of orientation. Brain Res. Bull..

[20] Scilingo EP, Bianchi M, Grioli G, Bicchi A (2010). Rendering softness: Integration of kinesthetic and cutaneous information in a haptic device. Haptics, IEEE Trans. on.

[21] Park J, Oh Y, Tan HZ (2018). Effect of cutaneous feedback on the perceived hardness of a virtual object. IEEE Trans. Haptics.

[22] van Beers RJ, Sittig AC, van Der Gon JJD (1999). Integration of proprioceptive and visual position-information: An experimentally supported model. J. Neurophysiol..

[23] Ernst MO, Banks MS (2002). Humans integrate visual and haptic information in a statistically optimal fashion. Nat..

[24] Chancel M, Blanchard C, Guerraz M, Montagnini A, Kavounoudias A (2016). Optimal visuotactile integration for velocity discrimination of self-hand movements. J. Neurophysiol..

[25] Hillis JM, Watt SJ, Landy MS, Banks MS (2004). Slant from texture and disparity cues: Optimal cue combination. J. Vis..

[26] Battaglia PW, Jacobs RA, Aslin RN (2003). Bayesian integration of visual and auditory signals for spatial localization. Josa a.

[27] Noel J-P, Serino A, Wallace MT (2019). Increased neural strength and reliability to audiovisual stimuli at the boundary of peripersonal space. J. Cognit. Neurosci..

[28] Kurita, Y., Yonezawa, S., Ikeda, A. & Ogasawara, T. Weight and friction display device by controlling the slip condition of a fingertip. In *2011 IEEE/RSJ International Conference on Intelligent Robots and Systems*, 2127–2132 (IEEE, 2011).

[29] Quek ZF, Schorr SB, Nisky I, Okamura AM, Provancher WR (2014). Augmentation of stiffness perception with a 1-degree-of-freedom skin stretch device. IEEE Trans. Human-Machine Syst..

[30] Park J, Han I, Lee W (2019). Effect of haptic feedback on the perceived size of a virtual object. IEEE Access..

[31] Collins DF, Refshauge KM, Gandevia SC (2000). Sensory integration in the perception of movements at the human metacarpophalangeal joint. J. Physiol..

[32] Bianchi, M. *et al*. Tactile slip and hand displacement: Bending hand motion with tactile illusions. In *World Haptics Conference (WHC), IEEE*, 96–100 (IEEE) (2017).

[33] Jenmalm P, Schmitz C, Forssberg H, Ehrsson HH (2006). Lighter or heavier than predicted: neural correlates of corrective mechanisms during erroneously programmed lifts. J. Neurosci..

[34] Flanagan JR, Bittner JP, Johansson RS (2008). Experience can change distinct size-weight priors engaged in lifting objects and judging their weights. Curr. Biol..

[35] Baugh LA, Kao M, Johansson RS, Flanagan JR (2012). Material evidence: Interaction of well-learned priors and sensorimotor memory when lifting objects. J. Neurophysiol..

[36] Kinoshita H, Bäckström L, Flanagan JR, Johansson RS (1997). Tangential torque effects on the control of grip forces when holding objects with a precision grip. J. Neurophysiol..

[37] Wing AM, Lederman SJ (1998). Anticipatory load torques produced by voluntary movements. J. Exp. Psychology: Hum. Percept. Perform..

[38] Goodwin AW, Jenmalm P, Johansson RS (1998). Control of grip force when tilting objects: effect of curvature of grasped surfaces and applied tangential torque. J. Neurosci..

[39] Quek ZF, Schorr SB, Nisky I, Provancher WR, Okamura AM (2015). Sensory substitution and augmentation using 3-degree-of-freedom skin deformation feedback. IEEE Trans. haptics.

[40] Girard A (2016). Haptip: Displaying haptic shear forces at the fingertips for multi-finger interaction in virtual environments. Front. ICT.

[41] Schorr SB, Okamura AM (2017). Three-dimensional skin deformation as force substitution: Wearable device design and performance during haptic exploration of virtual environments. IEEE Trans. haptics.

[42] Choi, I., Culbertson, H., Miller, M. R., Olwal, A. & Follmer, S. Grabity: A wearable haptic interface for simulating weight and grasping in virtual reality. In *Proceedings of the 30th Annual ACM Symposium on User Interface Software and Technology*, 119–130 (ACM, 2017).

[43] Zilles CB, Salisbury JK (1995). A constraint-based god-object method. haptic Disp..

[44] Ruspini D, Khatib O (2001). Haptic display for human interaction with virtual dynamic environments. J. Robotic Syst..

[45] Lunzman S, Kennedy D, Miller S (2008). Physical modeling of mechanical friction in simulink. Matlab Dig..

[46] Wojtyra M (2017). Modeling of static friction in closed-loop kinematic chains−uniqueness and parametric sensitivity problems. Multibody Syst. Dyn..

[47] Popov, V. L. *Contact mechanics and friction* (Springer, 2010).

[48] Olsson H, Åström KJ, De Wit CC, Gäfvert M, Lischinsky P (1998). Friction models and friction compensation. Eur. J. Control..

[49] Levitt H (1971). Transformed up-down methods in psychoacoustics. J. Acoustical Soc. Am..

[50] Barrea A, Delhaye BP, Lefèvre P, Thonnard J-L (2018). Perception of partial slips under tangential loading of the fingertip. Sci. Rep..

[51] Srinivasan MA, Whitehouse J, LaMotte RH (1990). Tactile detection of slip: surface microgeometry and peripheral neural codes. J. Neurophysiol..

