# Publisher Correction: Effect of Cutaneous Feedback on the Perception of Virtual Object Weight during Manipulation

**DOI:** 10.1038/s41598-020-60911-9

**Published:** 2020-02-28

**Authors:** Jaeyoung Park, Bukun Son, Ilhwan Han, Woochan Lee

**Affiliations:** 1 Korea Institute of Science and Technology, Robotics and Media Institute, Seoul, 02792 South Korea; 2 Seoul National University, Department of Mechanical Engineering, Seoul, 08826 South Korea; 3 Incheon National University, Department of Electrical Engineering, Incheon, 22012 South Korea

Correction to: *Scientific Reports* 10.1038/s41598-020-58247-5, published online 28 January 2020

The original version of this Article contained errors in the Abstract.

“Haptic interface technologies for virtual reality applica have been developed to increase the reality and manipulability of a virtual object by creating a diverse tactile sensation.”

now reads:

“Haptic interface technologies for virtual reality applications have been developed to increase the reality and manipulability of a virtual object by creating a diverse tactile sensation.”

“The results also indicate that the contact force applied to a virtual object during manipulation can be a function of the perceived object weight p = 0.005 for Experiment 1 and p = 0.2 for Experiment 2.”

now reads:

“The results also indicate that the contact force applied to a virtual object during manipulation can be a function of the perceived object weight (p = 0.005 for Experiment 1 and p = 0.02 for Experiment 2).”

These errors have now been corrected in the PDF and HTML versions of the Article.

